# Diverging Trends in Age at First Myocardial Infarction: Evidence from Two German Population-Based Studies

**DOI:** 10.1038/s41598-020-66291-4

**Published:** 2020-06-15

**Authors:** Johannes Beller, Johann Bauersachs, Andreas Schäfer, Lars Schwettmann, Margit Heier, Annette Peters, Christa Meisinger, Siegfried Geyer

**Affiliations:** 1 Hannover Medical School, Medical Sociology Unit, Hannover, Germany; 2 Hannover Medical School, Department of Cardiology and Angiology, Hannover, Germany; 3 Helmholtz Zentrum München, Institute of Health Economics and Health Care Management, Neuherberg, Germany; 4 Martin Luther University Halle-Wittenberg, Department of Economics, Halle-Wittenberg, Germany; 5 Helmholtz Zentrum München, Institute of Epidemiology, Neuherberg, Germany

**Keywords:** Cardiology, Epidemiology

## Abstract

Little is known about trends in the age of onset of first myocardial infarction. Thus, we examined trends in the age of onset distribution of first myocardial infarction using two population-based datasets from Germany. First, we used German claims data based on an annual case number of approximately 2 million women and men covering the period from 2006 to 2016. Second, we used data from the KORA (Cooperative Health Research in the Region of Augsburg) Myocardial Infarction Registry covering the period from 2000–2016. Analyses were performed by means of quantile regression to estimate trends across the whole distribution of age of onset. Overall, *N*_*Sample 1*_ = 69627 and *N*_*Sample 2*_ = 9954 first myocardial infarctions were observed. In both samples, we found highly heterogeneous trends in age of onset. In men, we consistently found that age of onset increased before 50 and after 70 but decreased within this age bracket. For women, on the other hand, we consistently found that age of onset decreased for first myocardial infarctions before 70 but increased slightly or remained relatively stable thereafter. Therefore, late myocardial infarctions tended to occur later in life, while regular myocardial infarctions tended to occur earlier. These results suggest that in myocardial infarction, both morbidity compression and morbidity expansion might have occurred at the same time but for different parts of the age at onset distribution.

## Introduction

Myocardial infarction (MI) is one major contributor to general morbidity. Most studies on MI occurrence have focused on disease rates, and MI rates in industrial countries have been observed to be decreasing since the 1970s^1–3^. Age of onset has been suggested as an additional epidemiological indicator that has been successfully used in research on diseases^4–7^: For example, it has been examined how the age at onset of stroke has changed over time, how the age at onset of depression has changed over time, how the age at onset of diabetes has changed over time, and how the age at onset of breast cancer has changed over time^4,8–10^. However, there is not much evidence regarding trends in age at onset in myocardial infarction (MI)^11^: An American study used Medicare data from 1999 to 2009^12^. Based on 5258 MI cases, the mean age at onset increased over the observation period. Another US-based study with male military personnel reported that the mean age at onset increased by 10 years over a period of 100 years^13^. These few studies, however, only considered changes in mean age at the upper end of the age scale, thus assuming that such changes may only occur at the upper end of the age distribution.

A shift in the mean age of MI onset in a population is also in accordance with Fries’ hypothesis of morbidity compression^14^, postulating that by shifting age at onset of disease and disability upward, shorter time periods will be spent in states of impaired health, and a healthy lifetime will be prolonged. However, the compression hypothesis does not make assumptions about the periods of life below the upper end and might thus paint an incomplete picture about population-based morbidity trends. Previous studies suggested that health in older adults seems to be improving the most, while health in comparatively younger age groups seems to be improving less or even deteriorating. For example, while some studies found that cardiovascular risk factors improved over time in Germany, others indicated that this seems to mostly apply to older but not middle-aged adults^15,16^. In particular, the increasing trends in diabetes and obesity among younger adults imply that while MI morbidity might decrease in older adults, it might actually increase in comparatively younger age groups^17–19^.

Thus, in the following, we analyse whether changes in age at MI onset occur only at the upper end, as suggested in the earlier studies cited above, or whether such changes can also be found in younger age groups. Analysing the age at onset is important because there exists only insufficient evidence regarding the age at onset in MI and because the age at onset is central to judgements about the existence or absence of a compression of morbidity in myocardial infarction^20^. The analyses will be performed with two large population-based datasets originating from two geographically distant regions with different social structures but covering overlapping age groups and time periods. This makes it possible to examine whether the same or comparable substantive results can be obtained and considered to validate each other, thus lending higher credibility to our findings. We ask: How has the age of onset of myocardial infarction changed over time?

## Methods

### Study populations

*AOK*. The first dataset consisted of pseudonymized claims data from the AOK Niedersachsen (Local Statutory Health Insurance of Lower Saxony) covering the years 2006 to 2015 with an annual population of >2 million insured women and men aged 18 years and older. The data were collected for accounting purposes and comprised basic socio-demographic information and hospital diagnoses. A detailed account of the dataset was published in an earlier paper^11^. As the proportion of elderly insured and thus the age at onset of diseases increase over time, a sampling procedure was applied to control for this demographic ageing: Random samples of the whole population for all age groups were drawn for every calendar year in such a way that the age distribution of the underlying population is held constant across the years. Then, in this age-standardized population, all new cases with admissions of first MI with ICD-diagnoses ICD-10: I21.0 to I21.9 were used. All analyses were performed with this dataset of first MI diagnoses that controlled for a changing age structure in the population. Due to the large sample size, additional analyses could also be conducted to explore trends in ST-segment elevation myocardial infarctions (STEMIs; I21.0– I21.3) and non-ST-segment elevation myocardial infarctions (NSTEMIs; I21.4).

*KORA*. The second dataset used data from the population-based KORA (Cooperative Health Research in the Region of Augsburg) Myocardial Infarction Registry, which was implemented as part of the WHO-MONICA (Monitoring Trends and Determinants in Cardiovascular Disease) project^21–23^. Since 1984, all cases of nonfatal MI and coronary deaths in the German region of Augsburg (the city of Augsburg and the counties Aichach-Friedberg and Augsburg) have been continuously registered for 25–74-year-olds. Most MI cases in the sample were recruited at the region’s major hospital, the university hospital of Augsburg. Additionally, information from death certificates was collected by the three public health departments in the study region. For the present analysis, all adults in the study region with a first MI during an observation period beginning in 2000 were included, while patients with a history of previous MI were excluded. Thus, the KORA sample consists of 9954 adults with a first MI aged 25 to 74 years at the time of MI covering the time from 2000 to 2016. The participants gave written informed consent, and the data collection was approved by the Ethics Committee of the Bavarian Medical Association.

### Data analysis

The analyses were performed by means of quantile regression^24^ to obtain a better understanding of potentially heterogeneous trends by examining changes in age at MI onset within defined age groups, in the present case within the quantiles of the age at onset distributions of the two datasets considered. By using quantile regression, it is possible not only to calculate average trends but also to study how the whole distribution of age at onset may have changed over time. For example, it might be expected that different parts of the population may exhibit different trends, with early-onset MI occurring earlier and late-onset MI occurring later. In this case, average trends may not accurately reflect the underlying mechanisms of change, and analyses might lead to biased conclusions. Quantile regression also avoids several flaws that arise from the use of stratified samples. One might argue that separate regression analyses could be run on age-stratified subsamples of the population that exhibit, for example, early and late onset of MIs. However, segmenting the population in this way results in smaller sample sizes for each stratified regression analysis and may be susceptible to sample selection bias. In contrast, quantile regression uses weights to obtain regression estimates that correspond to trends of different parts of the distribution and has been shown to be robust to outliers and non-normality. Therefore, employing quantile regression increases the power to detect differential trends and minimizes potential bias. In accordance with these benefits, quantile regression has been extensively used across scientific disciplines to study heterogeneous effects^25–28^.

In the first step, descriptive statistics were performed after dividing the age distribution into nine quantiles (10%, 20%, 30%, 40%, 50%, 60%, 70%, 80%, 90%). Then, quantile regression was used to determine the trends of age at onset for each age quantile. All quantile regressions were controlled for average population age to control for demographic ageing. Quantile regression directly estimates the quantiles across the distribution of age at onset, and it allows a more complete view of existing trends. Finally, we conducted the joint test for equality of slopes that tests whether the regression coefficients significantly differ across quantiles. All analyses were performed separately for women and men. The statistical package R (version 3.5.2) was used for all statistical analyses.

In summary, instead of relying on the accuracy of an “average trend”, quantile regression permits us to examine potentially heterogeneous trends for the whole age distribution of MI onsets. Thus, we examined how the whole distribution of age at onset of myocardial infarction has changed over time. Therefore, this study aims to provide (a) evidence for similar or heterogeneous trends of age at first myocardial infarction, (b) an empirical basis for classifying the onset of myocardial infarctions as early, average or late, and (c) to derive theoretical implications for the concept of morbidity compression. All methods were performed in accordance with the relevant guidelines and regulations.

## Results

### AOK claims data

**Figure 1:**
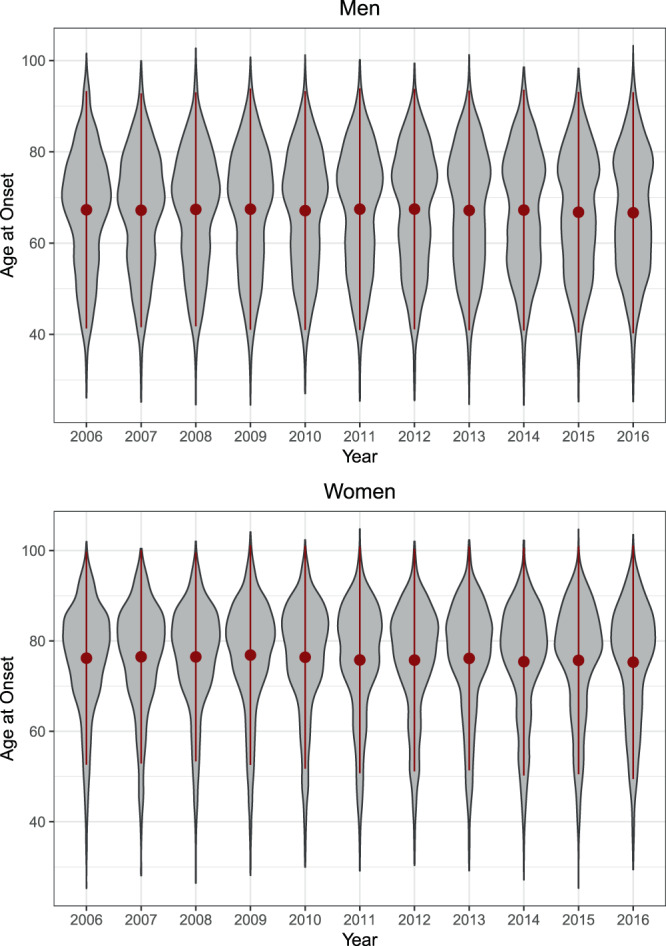
Descriptive trends in age at first myocardial infarction across the lifespan over time via Violin plots (AOK; N = 69627). Red points and lines depict the mean age at onset and the standard deviation of age at onset.

**Figure 2:**
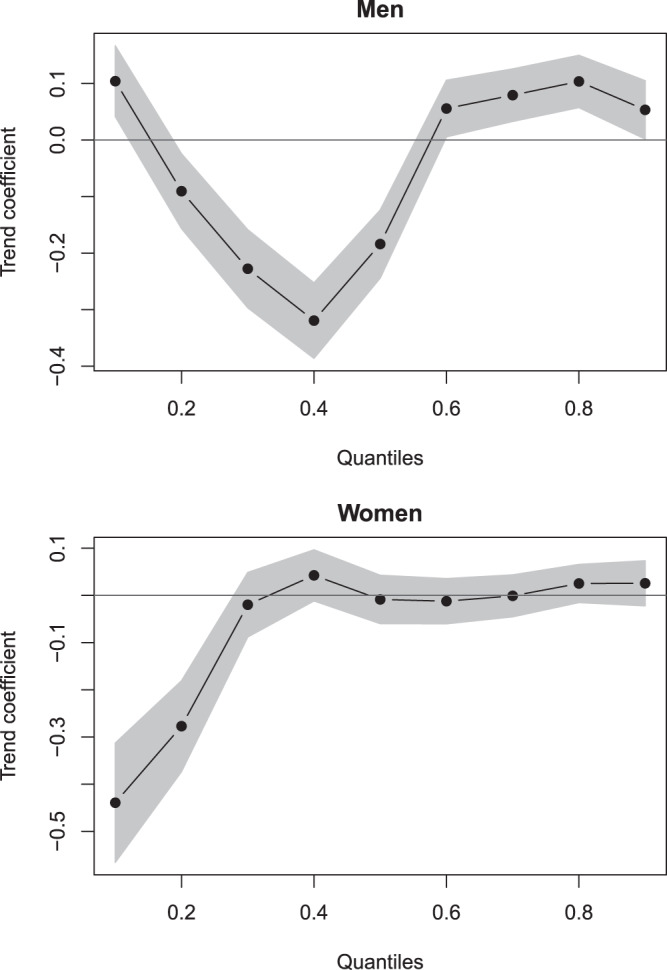
Effects of time on age at first myocardial infarction across quantiles (AOK; N = 69627). Shaded areas depict 95% confidence intervals so that those coefficients with shaded areas not encompassing zero can be considered statistically significant.

Descriptive trends in the development of first MIs are displayed in Tables 1 and 2 and visualized in Fig. 1. Overall, *N* = 69627 first MIs were observed, *N* = 41812 in men and *N* = 27815 in women. The number of cases of MI slightly decreased over time, with n = 6425 MIs in 2006 and n = 5832 MIs in 2016. In men, the mean age at first MI slightly decreased from 67.3 (in 2006) to 66.6 (in 2016). However, wide differences in trends over the whole population emerged (Fig. 1): In the 10% quantile, age at onset (corresponding approximately to age at first MI < 50) increased, while in the 20%-50% quantiles (approx. 50 < age > 70), it decreased. Then, age of onset increased again for the 60%-90% quantiles (approx. age > 70). Via quantile regression, we found that all described trends were significantly replicated, as seen in Fig. 2. Onset ages within the 10%, 60%, 70%, 80%, and 90% quantiles significantly increased over time, while within the 20%, 30%, 40%, and 50% quantiles, onset ages significantly decreased over time. The joint test for equality of slopes was significant and confirmed the heterogeneous trends across quantiles, p < 0.001. Thus, the age of MI onset decreased over time in men between 50 and 70 years of age but increased in men younger than 50 and older than 70.

**Table 1:** Characteristics of the two study populations.

Year	Men		Women		Sex Ratio
	# Cases	Average age at onset	# Cases	Average age at onset	
**Claims Data (AOK)**					
2006	3661	67.29	2764	76.19	1.32
2007	3695	67.19	2675	76.49	1.38
2008	3685	67.38	2581	76.46	1.43
2009	3552	67.43	2492	76.87	1.43
2010	3554	67.11	2432	76.39	1.46
2011	3999	67.43	2666	75.78	1.50
2012	4082	67.45	2719	75.75	1.50
2013	4017	67.15	2537	76.14	1.58
2014	3973	67.23	2427	75.41	1.64
2015	3909	66.76	2375	75.71	1.65
2016	3685	66.64	2147	75.30	1.72
**Registry Data (KORA)**					
2000	449	60.32	152	65.03	2.95
2001	464	60.37	170	63.56	2.73
2002	494	59.81	151	63.83	3.27
2003	457	60.65	168	64.24	2.72
2004	457	60.11	139	65.96	3.29
2005	500	61.02	179	62.71	2.79
2006	431	60.57	150	63.51	2.87
2007	395	59.61	145	63.41	2.72
2008	419	60.54	140	64.98	2.99
2009	423	60.75	150	63.05	2.82
2010	448	60.07	161	62.72	2.78
2011	438	59.90	158	65.16	2.77
2012	429	60.71	130	62.23	3.30
2013	442	61.00	149	63.79	2.97
2014	422	60.25	138	61.83	3.06
2015	405	59.96	126	61.83	3.21
2016	360	60.08	115	62.97	3.13

**Table 2:** Age at First Myocardial Infarction in AOK (N = 69627) by quantiles.

Year	n	M	SD	Quantiles								
				10%	20%	30%	40%	50%	60%	70%	80%	90%
**Men (N** = **41812)**												
2006	3661	67.29	13.00	48.56	55.29	61.14	65.81	68.99	71.72	74.85	78.27	83.38
2007	3695	67.19	12.80	48.99	55.30	60.20	65.37	68.81	71.70	75.08	78.51	82.93
2008	3685	67.38	12.81	49.07	55.37	60.23	65.73	69.19	72.16	75.17	78.65	83.06
2009	3552	67.43	13.20	48.59	54.78	60.00	65.52	69.46	72.50	75.79	79.17	83.54
2010	3554	67.11	13.07	48.76	54.54	59.59	64.79	69.09	72.11	75.27	79.06	83.27
2011	3999	67.43	13.23	48.64	54.52	59.67	64.65	69.70	72.89	75.98	79.42	83.69
2012	4082	67.45	13.16	49.25	54.65	59.84	64.22	69.46	72.98	75.96	79.55	83.60
2013	4017	67.15	13.13	49.11	54.57	58.97	63.56	68.36	72.71	75.83	79.40	83.89
2014	3973	67.23	13.18	49.88	54.80	58.84	63.30	68.08	72.82	75.97	79.43	83.93
2015	3909	66.76	13.17	49.13	54.12	58.84	63.13	67.27	72.12	75.49	79.08	83.40
2016	3685	66.64	13.19	49.51	54.41	58.47	62.41	66.47	71.43	75.73	79.12	83.31
**Women (N** = **27815)**												
2006	2764	76.19	11.78	59.74	67.81	71.83	75.38	78.20	80.65	83.24	85.72	89.26
2007	2675	76.49	11.80	59.79	68.12	72.10	75.83	78.73	81.17	83.68	86.02	89.24
2008	2581	76.46	11.55	59.93	68.92	72.55	75.73	78.53	80.88	83.28	85.88	88.73
2009	2492	76.87	12.15	59.33	69.02	73.12	76.27	79.27	81.57	83.97	86.48	89.61
2010	2432	76.39	12.32	58.40	68.33	72.31	75.65	78.90	81.54	84.12	86.58	89.38
2011	2666	75.78	12.52	57.48	66.33	72.07	75.12	78.01	81.20	83.56	86.44	89.31
2012	2719	75.75	12.30	56.71	66.59	72.26	75.54	78.21	80.97	83.36	85.74	89.03
2013	2537	76.14	12.38	57.56	66.56	72.66	75.85	78.48	81.25	83.67	86.39	89.62
2014	2427	75.41	12.59	55.47	64.90	72.08	75.47	78.18	80.52	83.17	85.87	88.83
2015	2375	75.71	12.59	56.18	65.24	71.89	76.07	78.31	80.77	83.43	86.24	89.73
2016	2147	75.30	12.91	56.37	63.87	70.25	75.35	78.23	80.73	83.45	86.11	89.55

In women, the mean age at first MI also slightly decreased from 76.2 (in 2006) to 75.3 (in 2016). Again, wide differences across the distribution emerged (Fig. 1): Within the 10%, 20% and 30% quantiles, age at onset decreased (approx. age < 72), while within the 40%-90% quantiles, it seemed to marginally increase or remained relatively stable (approx. age of onset > 72). Via quantile regression, we found that only the decreasing trends within the 10% and 20% quantiles (approx. age < 70) were significant, as displayed in Fig. 2. The joint test for equality of slopes was significant and confirmed the heterogeneous trends across quantiles, p < 0.001. Therefore, the age of onset in women decreased over time for first MIs before the age of 70 but remained relatively stable thereafter. Additional analyses, summarized in the Appendix, suggest that the age of onset of ST-segment elevation myocardial infarctions (STEMIs) mostly decreased, while differential trends across the lifespan were mostly observed in non-ST-segment elevation myocardial infarctions (NSTEMIs).

### KORA

**Figure 3:**
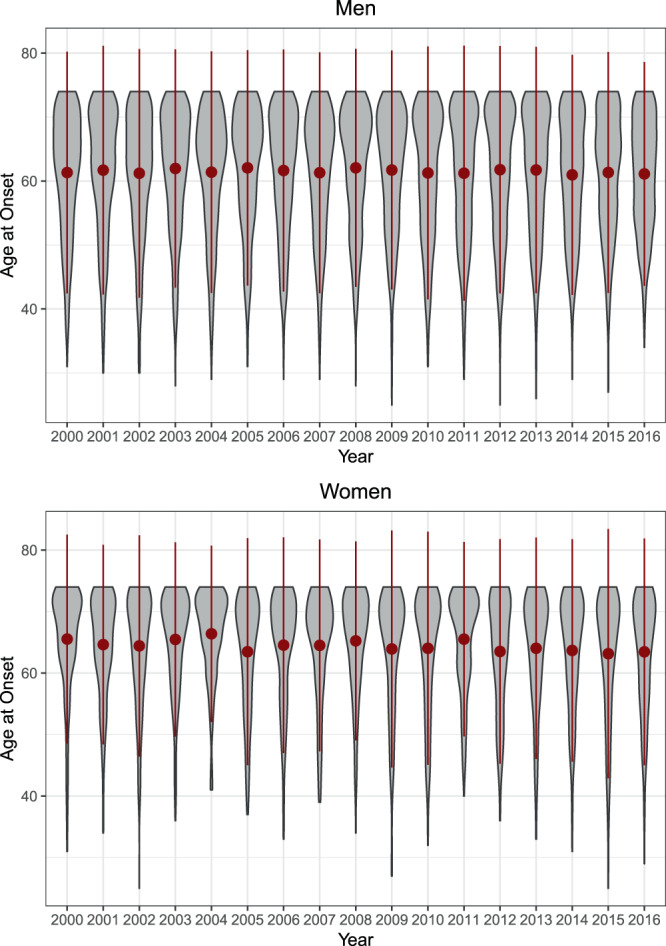
Descriptive trends in age at first myocardial infarction across the lifespan over time via Violin plots (KORA; N = 9954). Thicker areas represent more MIs. Red points and lines depict the mean age at onset and the standard deviation of age at onset.

Descriptive trends in age at first MI are displayed in Table 1 and Table 3 and visualized in Fig. 3. Overall, N = 9954 first myocardial infarctions and coronary deaths were recorded, N = 7433 in men and N = 2521 in women. The number of cases of MI slightly decreased over time, with n = 601 MIs in 2000 and n = 475 MIs in 2016.

**Table 3:** Age at First Myocardial Infarction in KORA (N = 9954) by age quantiles.

Year	n	M	SD	Age quantiles								
				10%	20%	30%	40%	50%	60%	70%	80%	90%
**Men (N** = **7433)**												
2000	449	60.32	9.89	46.00	51.00	55.00	59.00	62.00	65.00	67.00	70.00	72.00
2001	464	60.37	9.92	46.00	51.00	56.00	59.20	62.00	64.80	67.00	70.00	72.00
2002	494	59.81	9.70	46.00	52.00	55.00	59.00	62.00	63.00	66.00	69.00	72.00
2003	457	60.65	9.75	46.60	52.00	56.00	59.00	62.00	65.00	68.00	70.00	72.00
2004	457	60.11	9.64	46.00	52.00	55.00	60.00	62.00	64.00	66.20	69.00	71.00
2005	500	61.02	9.36	47.00	53.00	57.00	60.00	63.00	65.00	67.00	69.00	72.00
2006	431	60.57	9.57	46.00	51.00	56.00	59.00	63.00	65.00	67.00	70.00	72.00
2007	395	59.61	9.82	46.00	51.00	55.00	58.00	61.00	64.40	67.00	69.00	71.00
2008	419	60.54	9.57	47.00	51.00	56.00	59.00	62.00	65.00	68.00	70.00	72.00
2009	423	60.75	9.62	48.00	52.00	55.60	59.00	63.00	66.00	68.00	70.00	72.00
2010	448	60.07	10.17	45.70	51.00	54.00	58.00	61.50	65.00	68.00	70.00	72.00
2011	438	59.90	10.05	46.00	50.00	54.00	57.00	61.00	64.00	67.00	71.00	73.00
2012	429	60.71	9.81	46.00	51.00	55.00	58.20	62.00	66.00	68.00	71.00	72.00
2013	442	61.00	9.99	47.00	52.00	56.00	60.00	62.00	65.00	68.00	71.00	73.00
2014	422	60.25	9.19	48.00	51.00	55.00	59.00	62.00	64.00	66.00	69.00	72.00
2015	405	59.96	9.82	47.00	51.00	54.00	57.00	61.00	64.00	67.00	69.00	72.60
2016	360	60.08	8.76	48.00	52.00	55.00	57.00	61.00	63.00	66.00	68.00	72.00
**Women (N** = **2521)**												
2000	152	65.03	8.67	54.00	60.00	63.00	65.00	67.00	69.00	71.00	72.00	73.00
2001	170	63.56	8.83	51.00	56.00	60.70	62.00	65.00	68.00	70.00	72.00	73.00
2002	151	63.83	9.07	49.00	58.00	61.00	64.00	66.00	69.00	70.00	72.00	73.00
2003	168	64.24	8.35	53.70	58.00	60.10	64.00	67.00	68.00	70.00	72.00	73.00
2004	139	65.96	7.16	57.00	60.00	64.00	65.20	68.00	69.80	71.00	72.00	73.00
2005	179	62.71	9.42	49.00	54.00	58.00	62.00	66.00	68.00	70.00	71.00	73.00
2006	150	63.51	9.50	49.90	55.00	60.00	65.00	66.00	68.00	69.30	72.00	74.00
2007	145	63.41	9.12	48.00	57.00	60.00	64.00	67.00	68.00	69.00	71.00	73.00
2008	140	64.98	7.96	53.90	59.00	61.70	65.00	67.00	69.00	70.00	72.20	73.00
2009	150	63.05	9.68	48.80	54.80	60.70	63.00	66.00	68.00	70.00	71.00	72.10
2010	161	62.72	10.16	46.00	53.00	58.00	63.00	67.00	68.00	70.00	72.00	73.00
2011	158	65.16	7.98	52.00	60.00	62.00	64.00	68.00	70.00	71.00	72.00	73.00
2012	130	62.23	9.38	48.00	52.00	58.00	62.00	64.50	66.00	69.00	72.00	73.00
2013	149	63.79	8.98	51.80	57.00	60.00	63.00	66.00	68.80	70.00	72.00	73.00
2014	138	61.83	9.55	49.00	53.00	58.00	60.80	64.00	67.00	69.00	71.00	72.30
2015	126	61.83	11.04	46.00	52.00	57.00	62.00	65.00	68.00	70.00	71.00	73.00
2016	115	62.97	9.54	48.40	55.80	59.00	62.00	65.00	67.00	71.00	72.00	73.00

**Figure 4:**
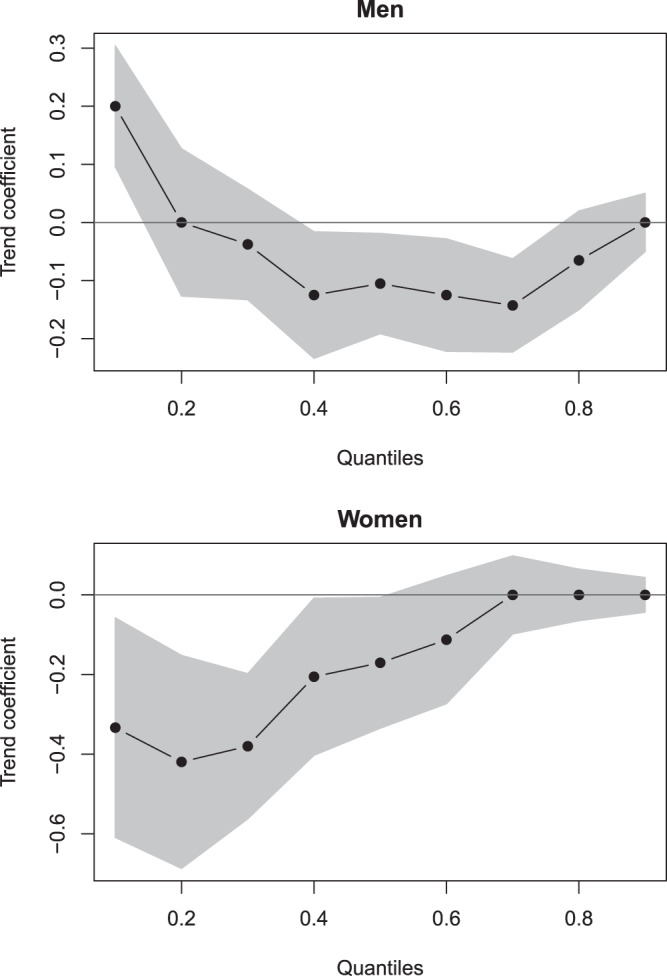
Effects of time on age at first myocardial infarction across quantiles (KORA; N = 9954). Shaded areas depict 95% confidence intervals, so that those coefficients with shaded areas not encompassing zero can be considered statistically significant.

In men, the mean age at first MI slightly decreased from 60.3 (in 2000) to 60.1 (in 2016). However, the trends in the population differed significantly (Fig. 3): In the 10% and 20% quantiles, age at onset increased (approx. age of onset < 52), while in the 40–80% quantiles, it decreased (approx. age > 55 and < 70). The age at onset in the 30% and 90% quantiles remained relatively constant. When controlling for the ageing population via quantile regression, the trends obtained with the health insurance data were generally replicated, as depicted in Fig. 4. However, only in the 10% quantile (approx. age of onset < 50) did age at onset significantly increase over time, and only in the 40%, 50%, 60%, and 70% quantiles did it significantly decrease over time (approx. age > 55 and <70). The joint test for equality of slopes was significant and thus confirmed the diverging trends across quantiles, p < 0.001. Therefore, in the KORA data, age of onset decreased for first MI in men between 55 and 70 years of age but increased in men younger than 50.

In women, the mean age at first MI decreased from 65.0 (in 2000) to 63.0 (in 2016). Again, there were differences across the distribution (Fig. 3): In the 10%–60% quantiles, age at onset decreased (approx. age of onset < 70), while there were no trends in the 70%, 80% and 90% quantiles (approx. age of onset > 70). When controlling for the ageing population by means of quantile regression, all described trends were replicated, as shown in Fig. 4. However, only the trends in the 10%, 20%, 30%, 40%, and 50% quantiles were statistically significant (age of onset < 68). The joint test for equality of slopes was significant and thus confirmed the diverging trends across quantiles, p = 0.008. Therefore, in the KORA data, age of onset decreased in women for first MI before age 68 but remained relatively stable thereafter.

## Discussion

We investigated how the age of onset of myocardial infarction has changed over time. We found that although the *mean* age of first myocardial infarction did not change much, there were strong diverging trends in the underlying *distribution* of age at first myocardial infarction. In men, there was a general tendency of age at first MI to increase beyond the age of 70 and below 50, suggesting a compression of MI-related morbidity in this group. Between 50 and 70 years, the mean age at onset, however, decreased, suggesting an expansion of MI-related morbidity. In women, the mean age of onset remained relatively stable or slightly increased for MIs occurring after age 70, while it decreased in the age groups below that age, suggesting an expansion of MI-related morbidity in this age group. However, this finding has to be interpreted against the backdrop of the largest share of onsets taking place far above the age of 70 in women. Therefore, the age at onset of MI showed divergent trends: late myocardial infarctions tended to occur later in life, while regular myocardial infarctions tended to occur earlier in the lifespan.

This is also in line with studies on trends in the age at onset regarding similar diseases. For example, in the case of stroke it has been reported that the mean age increased across time on average, as is the case in our study of MI^4^. Contrary to this trend that seems to be driven by improvements in older adults, other studies also reported that comparatively younger adults experienced an increasing burden of stroke^29^. Considering this converging evidence of health improvements in older subpopulations and health deteriorations in comparatively younger adults, it seems likely that shared risk factors between both stroke and MI have changed over time. For example, it seems likely that improvements in the lifestyle of older adults, such as increasing rates of physical activity, have been partly responsible for improved health of older adults. At the same time, trends such as the increasing prevalence of diabetes in younger adults might be responsible for a higher burden of cardiovascular disease in younger adults.

These results bear important implications regarding Fries’ hypothesis of morbidity compression^14^. There was no uniform development towards postponement of MI onset into higher age groups, as suggested by his hypothesis. Rather, the decreasing mean age at MI onset below 70 can be described as morbidity expansion^30^. Thus, our results point to two simultaneously existing trends of a compression of morbidity in higher age groups and an expansion of morbidity in younger age groups. The simultaneous existence of both these trends suggests that divergent trends in risk factors might have occurred. The existence of morbidity compression is often argued to result from improved primary prevention and changes in lifestyles in terms of more exercise, consumption of healthier food and decreased smoking rates^14,31–33^. There is mounting evidence, however, suggesting that while this might be the case in older adults, comparatively younger adults’ lifestyle might have also become unhealthier over time, which might explain the observed effects^18^.

Although we controlled for changing population age structures in our analyses, neither dataset includes longitudinal behavioural parameters that might permit us to verify potential explanations for the differential development of MI onset in our study populations. This particularly applies to the clinically relevant differentiation between MI with ST-segment elevation (STEMI) and MI with non-ST-segment elevation (NSTEMI). Both are different with respect to their aetiology as well as their short- and long-term prognosis^34^. The preliminary results reported in the Appendix suggest that the increase in age at onset in higher age groups is mainly visible in ST-elevation myocardial infarction and that the diverging trends are mainly observable in non-ST-elevation myocardial infarction. Thus, future studies should explore the potential mechanisms responsible for the differential trends of MI, possibly by investigating subtypes of MI. Nevertheless, our findings on diverging trends in MI for different age groups are well founded, as they were obtained from two datasets of different geographical origins and collected with different assessment modes.

If these results had been obtained from only a single database, they might be called into question, but they were derived from two very different datasets, lending more credibility to our findings. Although they covered similar age groups, they were collected for different purposes. In Germany, all residents must have health insurance, and in 2011, only 0.2% were uninsured. In Germany, statutory health insurance provides health care coverage for all residents below a fixed income threshold. Individuals above this threshold, government officials and many self-employed are allowed to be privately insured. Consequently, the first dataset consists of routine data for accounting, and the second dataset is register-based for research, depicting women and men from distant geographical areas. The KORA data continuously collected all cases of MI and coronary deaths for 25–74-year-olds in the German region of Augsburg, and the trends can thus be argued to be representative of the region of origin^21,22^. Claims data, on the other hand, provide large case numbers, but they are more selective with respect to socio-demographic composition. They include a higher proportion of individuals with low incomes and lower occupational positions, but the distributions of age and gender correspond to the population of Lower Saxony and Germany as a whole^35^.

Notwithstanding that our results were replicated in two samples and that they conform to trends in similar diseases as reported in the literature, future studies should still try to replicate and expand upon our results. For example, although the age at onset has been suggested and used in the literature as one important indicator of disease trends, there are other important metrics which should be investigated by future studies^6,36^. One avenue for future research could be the use of age-period-cohort models. e.g.^17,19,37^. Analysing how characteristics of MI are changing across age, time periods and birth cohorts might proof to be one effective way to independently replicate our results.

Thus, in summary, we investigated trends in age at onset in myocardial infarction using two population-based samples from Germany. We found that although there was not much change in the *mean* age at onset over time, there were large diverging trends across the *distribution* of age at first myocardial infarction. In both samples, late myocardial infarctions tended to occur later in life, while regular myocardial infarctions tended to occur earlier in the lifespan. This finding suggests the simultaneous existence of two contradicting trends: A compression of MI-related morbidity in higher age groups and an expansion of MI-related morbidity in comparatively younger age groups^37^. Future studies should explore the robustness of these findings and the mechanisms responsible for these trends.

## Supplementary information

APPENDIX. Additional Analyses.

## Data Availability

The data that support the findings of this study are available from the Helmholtz Zentrum München and the Allgemeine Ortskrankenkasse Niedersachsen, but restrictions apply to the availability of these data, which were used under license for the current study, and so are not publicly available. One can, however, apply for access to the data at the aforementioned institutions.
