# Supplementary material for: Diverging Trends in Age at First Myocardial Infarction: Evidence from Two German Population-Based Studies
Source: Sci Rep. 2020 Jun 15;10:9610. doi: 10.1038/s41598-020-66291-4 (PMC7296035; doi:10.1038/s41598-020-66291-4)

Diverging Trends in Age at First Myocardial Infarction: Evidence from Two German Population-Based Studies

Johannes Beller[1], Johann Bauersachs[2], Andreas Schäfer[2], Lars Schwettmann[3], Margit Heier[3], Annette Peters[3], Christa Meisinger[3], Siegfried Geyer[1]

1 Hannover Medical School, Medical Sociology Unit

2 Hannover Medical School, Department of Cardiology and Angiology

3 Helmholtz Zentrum München, Institute of Epidemiology

Contact Information:

Johannes Beller, Hannover Medical School, Medical Sociology, Carl-Neuberg-Str. 1, 30625 Hannover, Germany. Email: beller.johannes@mh-hannover.de

APPENDIX

Figure A1. Effects of time on age at first ST-Elevation Myocardial Infarction (STEMI) across quantiles (AOK). Shaded areas depict 95% confidence intervals so that those coefficients with shaded areas not encompassing zero can be considered statistically significant.

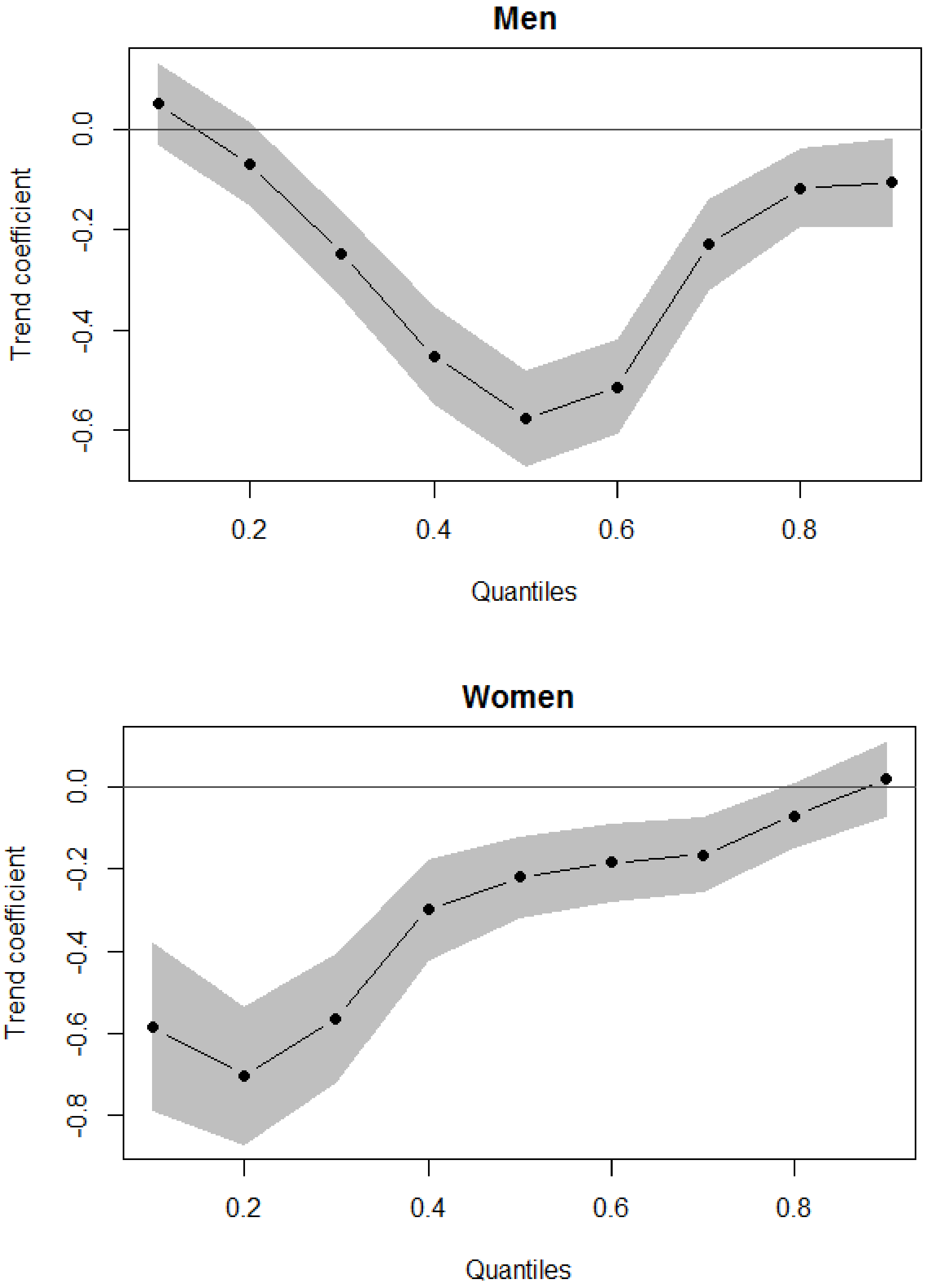

Figure A1. Effects of time on age at first Non-ST-Elevation Myocardial Infarction (NSTEMI) across quantiles (AOK). Shaded areas depict 95% confidence intervals so that those coefficients with shaded areas not encompassing zero can be considered statistically significant.

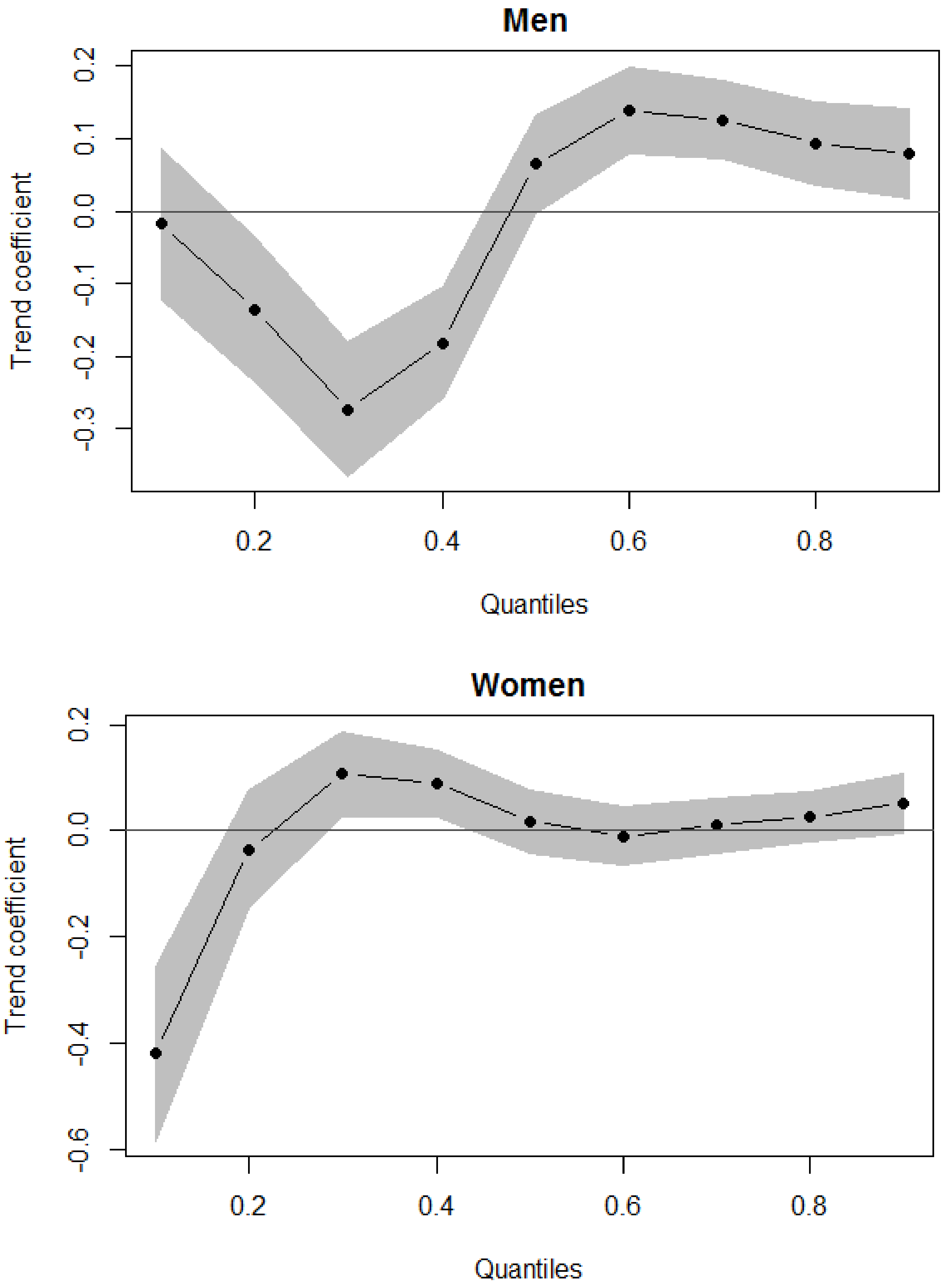

Supplement: Supplementary file 1 — APPENDIX. Additional Analyses. [file 41598_2020_66291_MOESM1_ESM.pdf]
